# High-Throughput Sequencing-Based Identification of miRNAs and Their Target mRNAs in Wheat Variety Qing Mai 6 Under Salt Stress Condition

**DOI:** 10.3389/fgene.2021.724527

**Published:** 2021-08-11

**Authors:** Xiaoyan He, Zhen Han, Huayan Yin, Fan Chen, Yihuan Dong, Lufei Zhang, Xiaoqing Lu, Jianbin Zeng, Wujun Ma, Ping Mu

**Affiliations:** ^1^College of Agronomy, Qingdao Agricultural University, Qingdao, China; ^2^State Agricultural Biotechnology Centre, College of Science, Health, Engineering and Education, Murdoch University, Perth, WA, Australia

**Keywords:** wheat, salt stress, transcriptome, microRNA, target gene, expression verification

## Abstract

Soil salinization is one of the major abiotic stresses that adversely affect the yield and quality of crops such as wheat, a leading cereal crop worldwide. Excavating the salt-tolerant genes and exploring the salt tolerance mechanism can help breeding salt-tolerant wheat varieties. Thus, it is essential to identify salt-tolerant wheat germplasm resources. In this study, we carried out a salt stress experiment using Qing Mai 6 (QM6), a salt-tolerant wheat variety, and sequenced the miRNAs and mRNAs. The differentially expressed miRNAs and mRNAs in salt stress conditions were compared with the control. As results, a total of eight salt-tolerance-related miRNAs and their corresponding 11 target mRNAs were identified. Further analysis revealed that QM6 enhances salt tolerance through increasing the expression level of genes related to stress resistance, antioxidation, nutrient absorption, and lipid metabolism balance, and the expression of these genes was regulated by the identified miRNAs. The resulting data provides a theoretical basis for future research studies on miRNAs and novel genes related to salt tolerance in wheat in order to develop genetically improved salt-tolerant wheat varieties.

## Introduction

Salt stress due to saline soil is one of the most severe abiotic stress that impedes crop growth and yield. It adversely affects more than 20% of the irrigated soil worldwide (Singh, 2020). More than 70 million hectares of the land area is affected by secondary saline-alkaline (FAO).^1^ Global climate change, immoderate irrigation, unsustainable development, and other factors are causing a continuous increase of salinized land (D’Odorico et al., 2013; Prăvălie et al., 2021). Soil salinization has severely affected agricultural land globally, posing a severe threat to agricultural development. Utilizing the saline land and controlling the land salinization level have become a global issue in relation to food security and agriculture sustainability (Li et al., 2014). Multiple studies have proven that cultivating and planting salt-tolerant crops is the most economical and effective way to utilizing saline-alkaline land for crop production (Galvani, 2007).

Wheat is one of the three major food crops worldwide and is a staple food for more than 30% of the world population that provides nearly 20% of the global energy consumption (FAO; see footnote 1). Its plantation accounts for about 17% of the total cultivated area worldwide, globally, wheat is one of the major crops being cultivated on saline-alkaline land, which plays an important role in managing and utilizing saline-alkaline soil. A continuous worsen in global warming, industrial pollution, lack of water resources, and abnormal irrigation has alarmingly increased soil salinization in the world’s leading wheat-producing areas, which has posed a serious threat to wheat production worldwide (Nadeem et al., 2020). In this study, we investigated the salt tolerance genes in a unique wheat genotype with an aim to improve wheat salt tolerance.

Recent advances in high-throughput sequencing technology have substantially increased the wheat genome data availability and promoted the investigative studies on wheat transcriptome and miRNAs (Kuang et al., 2019). The miRNAs are non-coding single-stranded small RNAs (18–24 nt) that are involved in post-transcriptional regulation by binding to their specific target mRNA (Singh et al., 2020). In plants, RNA polymerase II mediates the transcription of pri-miRNAs with cap structure and polyadenylate tail from endogenous miRNA genes. pri-miRNAs are then cleaved by Drosha/DGCR8 complex to form hairpin pre-miRNAs, which are exported to cytoplasm from the nucleus *via* Exportin-5 and Ran-GTP. In the cytoplasm, the Dicer enzyme converts this pre-miRNA to 20 bp double-stranded miRNAs. Under the RNA silencing complex (RISC), mature miRNAs regulate the target gene expression by inhibiting its translation or mediating its degradation. As shown in a previous study, miRNA plays a vital role in plant morphogenesis, growth regulation, hormone secretion, and signal transduction (Zhang and Unver, 2018). Additionally, miRNA-mediated post-transcriptional regulation is crucial to improving stress tolerance in crops (Pagano et al., 2021). However, regardless of its importance, there are few studies so far on miRNAs in salt-tolerance in wheat.

To investigate the salt-tolerance-related miRNAs and their target mRNAs in wheat and to explore their involvement in salt-tolerance, a salt-tolerant variety Qing Mai 6 (QM6) was analyzed under NaCl-induced salt stress in this study and led to identification of a set of differentially expressed miRNAs and mRNAs in the cultivar under salt stress. Target genes of differentially expressed miRNAs were predicted based on the differentially expressed mRNAs, and functional analysis of these target genes was performed. The biological processes and pathways associated with the differential miRNAs and their target mRNAs were identified. In addition, miRNA mediated salt-tolerance mechanism of QM6 was discussed. This study provides a reference for future studies on salt-tolerance-related miRNAs in wheat and unravels some candidate genes for genetic improvement of salt-tolerant wheat variety.

## Materials and Methods

### Hydroponic Experiment

This study uses two wheat genotypes, a cultivar QM6 and an old wheat line Chinese Spring (CS). QM6 is one of the widely cultivated wheat varieties in Shandong Province, one of China’s primary wheat-producing areas and is known as a salt-tolerant wheat variety, while CS is a salt-sensitive variety. QM6 and CS wheat seeds with full and uniform size were selected and sterilized with 75% alcohol for 5 min. Distilled water was used to rinse these wheat seeds 2–3 times. The sterilized wheat seeds were placed in petri dishes with two layers of wet test paper to germinate. After 7 days, the germinated wheat seeds were transferred to a 5 L black plastic bucket containing 1/5 Hoagland nutrient solution and placed in a controlled glasshouse, which was set to 25°C with a photoperiod of 12 h, a luminous flux of 3,000 Lx, and relative humidity of 65%. The nutrient solution was changed every 2–3 days during the seedling growth. The pH of the nutrient solution was set to 5.8–6.0 and was continuously aerated with pumps throughout the experiment. At two-leaf seedlings stage, half of the buckets were treated with 200 mM NaCl to induce salt stress. The plants without stress treatment were considered as control. Each treatment has three biological replicates. After 24 h, roots from the two treatments were quickly collected and cooled in liquid nitrogen and then stored in a refrigerator at −80°C for RNA extraction. The remaining plants underwent a further 2-week growth under stress or regular conditions, then all plants were collected and their dry weights of roots and shoots were measured after drying them for 48 h in an oven at 80°C.

### miRNA Library Construction and Sequencing

The total RNA was extracted using Takara MiniBEST Plant RNA Extraction Kit as per the manufacturer’s instruction. The concentration and purity of the extracted RNA were determined using NanoDrop ND1000 (NanoDrop Technologies, United States). RNA integrity was inspected using 1% agarose gel electrophoresis and Agilent 2100 (Agilent Technologies, United States). High-throughput sequencing of miRNA was performed by Annoroad Gene Technology Inc. (Beijing, China). Appropriate fragments were selected from the RNA samples that passed the quality test, and the 17–30 nt RNA fragments were enriched using gel separation technology. Connectors were added to the two ends of miRNA (a hydroxyl group at the 3' end and phosphate group at the 5' end) and reverse transcribed into cDNA. The sequencing library was constructed post-PCR amplification. The SE50 sequencing strategy was used to perform Illumina high-throughput sequencing for the constructed sequencing library (Hafner et al., 2008; Dillies et al., 2013).

### Prediction and Analysis of miRNA

The Illumina high-throughput sequencing was initially presented in an original image data file. After base calling, it was converted into raw reads using CASAVA v1.8.2 (Illumina Inc., 2011). The raw reads were spliced, low-quality reads were filtered, and fragments were selected using cutadapt v1.12^2^ to obtain clean reads for subsequent analysis (Martin, 2011). The clean reads were mapped to GenBank^3^ and Rfam^4^ databases to obtain annotation of non-coding RNAs. The interference of non-coding RNA, such as snoRNA, snRNA, tRNA, and rRNA, was eliminated through screening and then the selected reads were analyzed and aligned in miRbase v21.0^5^ according to the principle of most two mismatches (Kozomara and Griffiths-Jones, 2014). Clean reads that matched in miRBase v21.0 were termed as “known miRNAs” and the other reads were analyzed using miRDeep2 v0.01^6^ for “novel miRNAs” prediction.

The number of reads was firstly standardized for miRNA expression analysis (Transcripts Per Kilobase Million, TPKM). Then, the miRNA expression was calculated based on TPKM (Wagner et al., 2012). Finally, DESeq2 v1.20.0^7^ (Love et al., 2014) was used for differential expression analysis. The screening criteria for differential miRNA were false discovery rate (FDR) ≤ 0 and |log_2_FC| ≥ 1. FC represents fold change.

### mRNA Library Construction and Sequencing

The total root RNA from the three biological replicates of the two treatments was extracted using Takara MiniBEST Plant RNA Extraction Kit for sequencing library construction; thus, a total of six RNA samples were obtained. Agarose gel electrophoresis and NanoDrop were used to evaluate the RNA quality. The RNA concentration was determined by Qubit instrument (Thermo Fisher Scientific, Germany) and the RNA integrity was checked using Agilent 2100 Bioanalyzer (Agilent Technologies, United States).

Dynabeads Oligo (dT) was applied to enrich mRNA. Then, fragmentation buffer was added to break mRNA into short fragments. The first strand of cDNA was synthesized by random hexamers using short mRNA fragments as the template. The second strand of cDNA was synthesized after adding buffer, dNTPs, RNase H, and DNA Polymerase to tubes containing first strand of cDNA.

cDNA was purified using QiaQuick PCR Kit, and the EB buffer solution was used for cDNA elution. After terminal repair, base A was added to cDNA, followed by the addition of the sequencing adapters. Furthermore, agarose gel electrophoresis was used to recover the target size cDNA fragments for PCR amplification and RNA sequencing library construction. The constructed library was used for Illumina HiSeq sequencing.

### mRNA Sequencing Data Analysis

Quality evaluation was performed on the raw sequencing data. The adapters and the low-quality reads were removed to obtain clean reads. HISAT2 program^8^ was used to match clean reads with wheat reference genome (IWGSC v1.0) and to obtain annotation of clean reads with statistically analyzed (Kim et al., 2015). Based on the selected reference genome, the mapped reads were spliced using StringTie v1.3.4 (Pertea et al., 2015).^9^ After comparing the original genome annotation information, the uncommented transcriptional regions were identified as the novel transcripts and genes. The amino acid reads of novel genes were screened against the Pfam database^10^ using HMMER v3.2.1^11^ software to obtain the annotations of novel genes (Potter et al., 2018). The gene expression level was obtained through quantitative calculation of reads using FPKM (fragments per kilobase of transcript per million fragments mapped) method (Florea et al., 2013). The differentially expressed genes (DEGs) between the stressed and control samples were analyzed by Deseq2 v1.20.0 (see footnote 7) combined with statistical significance analysis to obtain values of *p*. Additionally, multiple tests for values of *p* were conducted based on FDR. Finally, genes with FDR < 0.05 and relative expression |log_2_FC| ≥ 2 were used to identify DEGs FC represents fold change.

Gene Ontology (GO) enrichment and KEGG analysis were performed for differentially expressed mRNAs using Blast and HMMER software. According to the differentially expressed miRNAs and mRNAs, psRobot v1.2^12^ was used to predict the target mRNAs of miRNAs (Wu et al., 2012).

### Verification of Expressions of miRNA and Target Genes by qRT-PCR

Qing Mai 6 salt-treated root samples were prepared with three biological replicates. RNA was extracted from these samples and its concentration and integrity were detected as mentioned above. Mir-XTM miRNA First-Strand Synthesis Kit (Takara, Japan) was used to reverse transcribe miRNA single-strand into cDNA. Single strand cDNA of mRNA was reverse transcribed using Evo M-MLV RT Kit with gDNA Clean for qPCR IIKit (AG, China). Subsequently, the fluorescent quantitative PCR of miRNAs and their target mRNAs was performed on a CFX96 PCR instrument (Bio-Rad, United States) using SYBR-Green fluorescent dye (Bio-Rad, United States). U6 and actin were used as an internal control to correct the expression value (Paolacci et al., 2009; Feng et al., 2012). The specific procedure of PCR was 40 cycles at 95°C and 30 s (95°C for 5 s and 60°C for 30 s). The dissolution curve program was 60–95°C, 5 s in each step, increasing by 0.5°C. The specificity of PCR primers was verified by the dissolution curve. Finally, the original expression value of fluorescent quantitative PCR was obtained, and the relative expression values of genes were calculated through the 2-ΔΔCq relative quantitative method. Each experiment was repeated three times. qRT-PCR primers were designed using the reads sequences (Supplementary Table 1). The primers used in this study are shown in Supplementary Table 2.

### Data Analysis

SPSS software was used to analyze the significant difference between the data through the Tukey test. Origin software was used to draw the column diagram.

## Results

### Determination of Salt Tolerance in QM6

**Figure 1:**
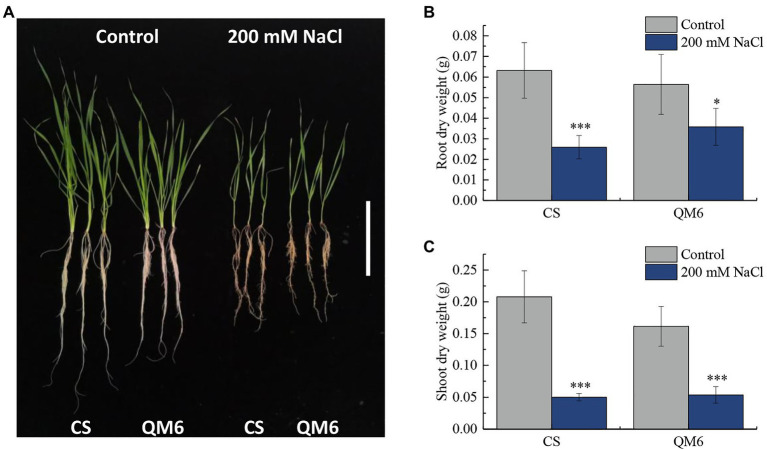
The Chinese Spring (CS) and Qing Mai 6 (QM6) phenotype under 200 mM NaCl-induced salt stress and control conditions. **(A)** The phenotypic difference of CS and QM6 in response to salt stress; bar = 5 cm. **(B)** The CS and QM6 root dry weights under 200 mM NaCl-induced salt stress and control conditions. **(C)** The CS and QM6 shoot dry weights under 200 mM NaCl-induced salt stress and control conditions. *n* = 3; ^*^p < 0.05; ^***^p < 0.001.

Qing Mai 6 and CS phenotypes did not vary significantly under the control condition. However, CS root and shoot growth were inhibited more significantly than that of QM6 under 200 mM NaCl treatment for 14 days (Figure 1A). Compared to the control, the dry weight of CS root decreased significantly by 59.03% under salt-stress, while the QM6 root only decreased by 36.56%, which is significantly less than that of CS (Figure 1B). For shoot dry weights, the CS and QM6 both decreased significantly by salt-stress, decreased by 75.85% in CS and 66.71% in QM6 (Figure 1C). These results suggest that the salt tolerance of QM6 was significantly higher than CS.

### Identification of QM6 miRNA

23,919,344 and 22,852,249 clean reads from QM6 roots under control and NaCl-induced salt stress conditions were obtained, respectively. Most reads were annotated into different categories (Table 1). rRNA preponderances of 13,629,988 (56.98%) and 11,586,708 (50.70%) reads were observed in the control and NaCl salt-treated root samples, respectively (Table 1). The known miRNA accounted for 0.62% (147,885) and novel miRNA accounted for 0.78% (185,934; Table 1) of the total reads from control conditions. However, under salt stress, the known miRNA accounted for 0.88% (202,005) while novel miRNA accounted for 1.62% (369,704; Table 1) of the total reads. These results showed the differences in miRNA expression between the control and NaCl-treated QM6 root samples.

**Table 1:** Summary of small RNA-seq data in QM6 under two treatments.

Treatment	Classification	Reads	Percentage (%)
Control	Clean reads	23,919,344	100
	Exon+	2,633,855	11.01
	Exon−	2,069,878	8.65
	Intron+	573,740	2.40
	Intron−	469,293	1.96
	Known miRNA	147,885	0.62
	Novel miRNA	185,934	0.78
	Repeat	28,766	0.12
	rRNA	13,629,988	56.98
	snRNA	354,419	1.48
	snoRNA	229,749	0.96
	tRNA	3,122,092	13.05
	Other	473,745	1.98
Salt stress	Clean reads	22,852,249	100
	Exon+	2,105,445	9.21
	Exon−	2,200,768	9.63
	Intron+	344,826	1.51
	Intron−	328,650	1.44
	Known miRNA	202,005	0.88
	Novel miRNA	369,704	1.62
	Repeat	17,575	0.08
	rRNA	11,586,708	50.70
	snRNA	302,457	1.32
	snoRNA	164,191	0.72
	tRNA	4,892,895	21.41
	Other	337,025	1.47

**Figure 2:**
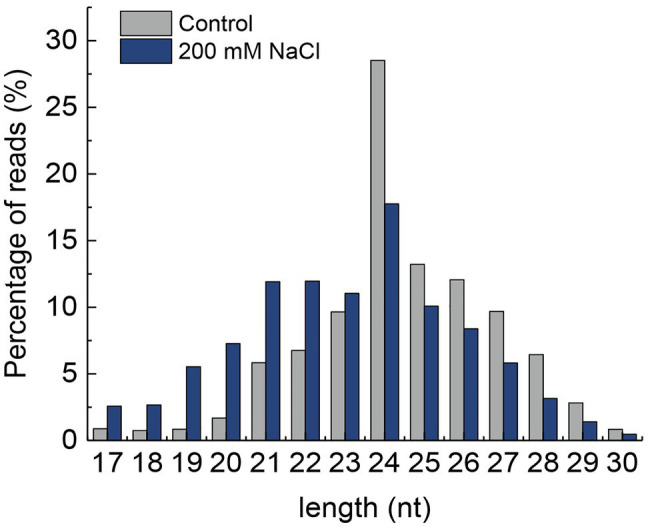
Length distribution of small RNAs in the QM6 roots under 200 mM NaCl-induced salt stress and control conditions. *Y*-axis, the percentage of small RNA reads.

The length distribution of miRNA showed that the number of reads from 24 to 30 nt was higher in control than that in NaCl-treated samples. However, the number of reads from 17 to 23 nt was higher in the control than that of the NaCl-treated samples. Moreover, the 24 nt nucleotide miRNA was found to be the most abundant in both control and NaCl-treated QM6 root samples (Figure 2). A total of 687 miRNAs were identified in both control and NaCl-treated QM6 root samples, of which 108 were known miRNAs (Supplementary Table 3) and 579 were novel miRNAs (Supplementary Table 4).

### Identification of QM6 mRNA Sequencing Data

A total of 224,115,086 clean reads from QM6 root samples were obtained after removing adapter and low-quality reads and then selecting fragment from the original transcriptome sequencing data. The clean bases were more than 9.50 Gb in all samples. Q30, the base mass value, was more than 92.86% and the GC content was 40–60% (Supplementary Table 5). In the control group, 67,583,952, 71,982,771, and 61,141,717 mapped reads were obtained, which account for 89.67, 89.95, and 89.63% of the total reads, respectively. In NaCl-treated samples, 60,915,291, 65,287,118, and 63,225,488 mapped reads were obtained, accounting for 84.72, 84.74, and 83.55% of the total reads, respectively (Table 2). The utilization rate of transcriptome data in control and NaCl-treated QM6 root samples was more than 83.55%, which met the requirement of the subsequent analysis. In the control group, reads matched to the unique position of the reference genome were 86.35, 86.73, and 86.42%, while in the NaCl-treated group, the unique mapped reads were 81.71, 81.76, and 80.58%. At least 44.21 and 41.28% of reads matched to the positive strand, and at least 44.41 and 41.42% of reads matched to the negative strand of the reference genome in the control and NaCl-treated QM6 root groups, respectively (Table 2).

**Table 2:** Summary of high throughput transcriptome sequencing of QM6 under two treatments.

Sample	Total reads	Mapped reads	Unique mapped reads	Multiple map reads	Reads map to “+”	Reads map to “−”
QM6 CK1	75,367,810	67,583,952 (89.67%)	65,082,516 (86.35%)	2,501,436 (3.32%)	33,318,887 (44.21%)	33,474,236 (44.41%)
QM6 CK2	80,024,874	71,982,771 (89.95%)	69,402,325 (86.73%)	2,580,446 (3.22%)	35,518,921 (44.38%)	35,664,950 (44.57%)
QM6 CK3	68,212,420	61,141,717 (89.63%)	58,948,101 (86.42%)	2,193,626 (3.22%)	30,174,820 (44.24%)	30,294,445 (44.41%)
QM6 T1	71,906,110	60,915,291 (84.72%)	58,753,740 (81.71%)	2,161,551 (3.01%)	30,100,486 (41.86%)	30,203,211 (42.00%)
QM6 T2	77,046,224	65,287,118 (84.74%)	62,993,212 (81.76%)	2,293,906 (2.98%)	32,255,771 (41.87%)	32,363,004 (42.00%)
QM6 T3	75,672,734	63,225,488 (83.55%)	60,974,788 (80.58%)	2,250,700 (2.97%)	31,240,150 (41.28%)	31,342,034 (41.42%)

### Identification of QM6 miRNA and mRNA in Response to Salt Stress

**Figure 3:**
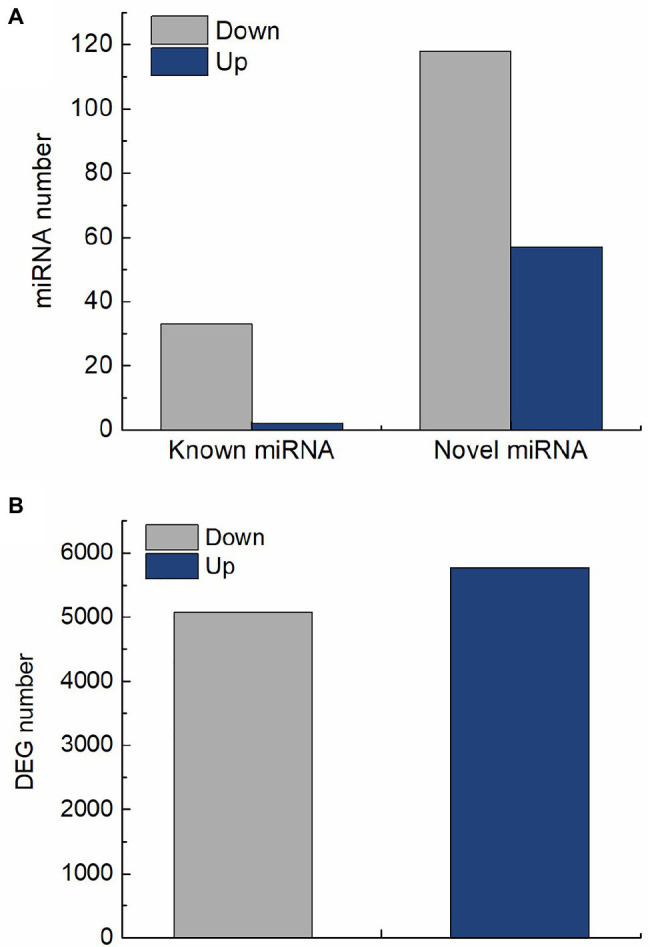
The number of differentially expressed miRNAs and genes in QM6 roots in response to salt stress. **(A)** The number of up-regulated and down-regulated known and novel miRNAs after 24 h of salt stress. **(B)** The number of up-regulated and down-regulated genes after 24 h of salt stress.

In this study, 210 differentially expressed miRNAs were identified in QM6 roots between control and salt stress conditions. Out of these, 35 were known miRNAs (Supplementary Table 6; Supplementary Figure 1) and 175 were novel miRNAs (Supplementary Table 7; Supplementary Figure 2). For the 35 known miRNAs, two were significantly up-regulated and 33 were significantly down-regulated (Figure 3A). Out of the 175 novel miRNAs, 57 were significantly up-regulated and 118 were significantly down-regulated in response to salt stress (Figure 3A). A total of 10,847 DEGs were identified in QM6 roots between control and salt stress conditions, of which 5,771 were up-regulated and 5,076 were down-regulated (Figure 3B; Supplementary Figure 3; Supplementary Table 8).

### Salt-Tolerance Related miRNAs and Their Target mRNAs

In order to explore the potential biological functions of differentially expressed miRNA in QM6 root samples under salt stress conditions, the differentially expressed miRNAs and mRNAs were analyzed. Target mRNAs of the differentially expressed miRNAs were predicted based on the obtained differentially expressed mRNAs. As results, eight miRNAs (seven known miRNA and one novel miRNA) and their corresponding 11 target mRNAs were determined (Table 3). The target mRNAs mainly included MYB-like gene, Cytochrome P450, POT family, NB-ARC domain protein, and GDSL-like lipase (Table 3). miRNAs including tae-miR1122a, tae-miR1131, tae-miR9774, and tae-miR9668-5p each had two target mRNAs while tae-miR319, tae-miR9674b-5p, tae-miR9666b-3p, and Novel_72 each had only one target mRNA (Table 3).

**Table 3:** Salt tolerance related miRNAs and their target genes in QM6.

miRNA	miRNA log_2_^FC^	Target gene ID	Target gene log_2_^FC^	Gene annotation
tae-miR1122a	−1.64	TraesCS3D02G020600	1.24	GDSL-like lipase
		TraesCS3B02G020700	2.48	Unknown
tae-miR1131	−1.84	TraesCS7B02G312500	1.19	POT family
		TraesCS7D02G136400	2.03	Cytochrome P450
tae-miR319	−1.53	TraesCS3A02G108000	1.52	MYB-like gene
tae-miR9774	−1.34	Triticum_aestivum_newGene_26286	1.09	NB-ARC domain
		Triticum_aestivum_newGene_26283	1.96	NB-ARC domain
tae-miR9674b-5p	−1.16	Triticum_aestivum_newGene_7847	1.07	Unknown
tae-miR9666b-3p	−3.41	TraesCS1B02G449200	2.58	MYB-like gene
tae-miR9668-5p	−1.69	TraesCS1A02G025500	2.65	NB-ARC domain
		TraesCS7A02G038900	2.78	NB-ARC domain
Novel_72	−8.43	TraesCS7B02G312500	1.19	POT family

### Expressions of Salt-Tolerance Related miRNAs and Their Target mRNAs

**Figure 4:**
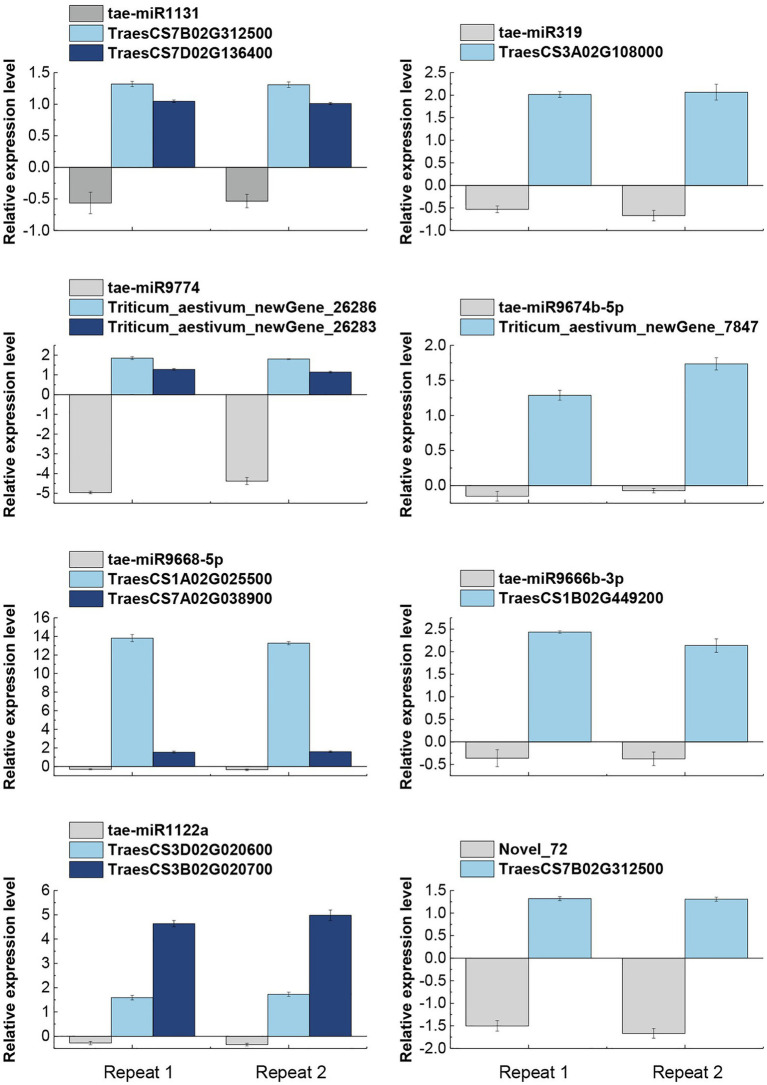
qRT-PCR based validation of miRNAs and their target genes expression pattern in response to salt stress. The expression data are means of three biological replicates and two technical repeats. tae-miR1131, tae-miR9774, tae-miR9668-5p, and tae-miR1122a miRNAs were found to have two target genes each, while tae-miR319, tae-miR9674b-5p, tae-miR9666b-3p, and Novel_72 miRNAs had only one target gene each.

All eight miRNAs and their 11 corresponding target mRNA were subjected to qRT-PCR based validation. The accuracy of miRNA and mRNA sequencing and target gene identification was also validated. qRT-PCR based on three biological replicates and at least two technical replicates showed that all miRNAs were down-regulated whereas their target mRNAs were up-regulated (Figure 4), which were highly consistent with the sequencing results. In conclusion, a negative correlation was observed between the expression levels of the eight verified miRNAs and their target genes. It demonstrated the reliability of the miRNA sequencing results and the identification of their target mRNAs.

## Discussion

miRNAs are a class of non-coding single-stranded small RNAs, which mediate multiple biological processes, including plant growth, development, and stress response by post-transcriptional or translational regulation of target genes (Kumar et al., 2017; Tang and Chu, 2017; Nair et al., 2019; Cui et al., 2020). As reported previously, miRNAs can improve salt tolerance in rice (Shen et al., 2017), barley (Kuang et al., 2019), *Arabidopsis thaliana* (Schommer et al., 2014), pearl chestnut (Harshraj et al., 2020), and other plants by regulating plant development and stress response. However, only few studies have been carried in wheat miRNAs related salt tolerance.

**Figure 5:**
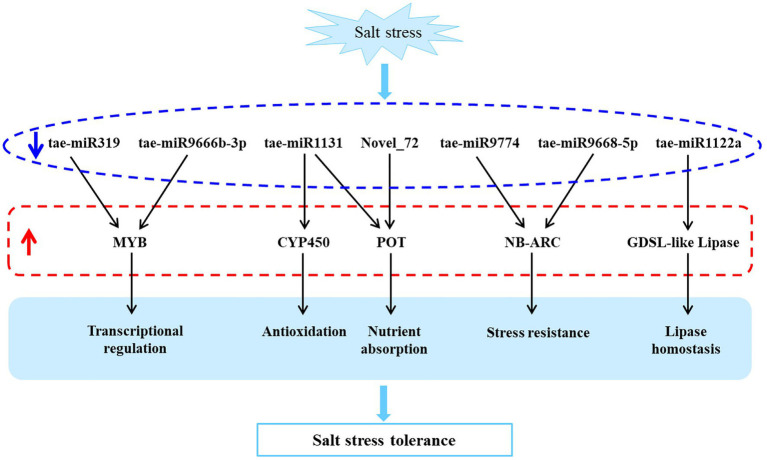
A predicted salt tolerant model of QM6 based on the differentially expressed miRNAs and their target genes. The blue arrow represents down-regulation and the red arrow represents up-regulation.

In this study, we investigated a salt-tolerant and widely cultivated wheat cultivar, QM6, using high-throughput sequencing and qRT-PCR based validation. And the rRNA reads accounted for less than 60% of total reads from plant RNA samples, indicating a good sequencing quality (Zhao et al., 2014). It led to the identification of eight differentially expressed miRNAs and their corresponding 11 target mRNAs involved in transcriptional regulation (MYB), oxidation resistance (CYP450), nutrient uptake (POT), stress tolerance (NB-ARC), lipid balance (GDSL-like lipase), and other biological processes, which can potentially be utilized to improve the salt tolerance of wheat (Figure 5).

### tae-miR319 and tae-miR9666b-3p Affected Salt Tolerance of Wheat by Regulating MYB Transcription Factor

miR319, an essential miRNA, plays a vital role in the growth and development, immune response, and abiotic stress in *A. thaliana*, barley, wheat, and other plants by regulating TCP, MYB transcription factor, and other miRNAs. *Arabidopsis thaliana* miR319a affects plant leaf development (Schommer et al., 2014) through TCP transcription factor regulation and plant root growth through MYB transcription factor regulation and *MYB33* gene transcription (Li et al., 2020). Barley miR319a regulates and participates in salt stress response by inhibiting the miR396e-mediated *GRF* gene pathway through *TCP4* expression (Kuang et al., 2019). Wheat miR319 is involved in regulating plant architecture, flowering, yield, and other important agronomic traits by targeting the *TaMYB34* gene (Jian et al., 2019). Furthermore, some members of the miR9666 family play a crucial role in abiotic stress response (Li et al., 2017). A previous study showed that tae-miR9666b miRNA is only expressed at the seedling stage of wheat (Han et al., 2014). However, no study has been carried in salt tolerance aspect about tae-miR9666b.

As one of the major transcription factors in plants, MYB plays a crucial role in plant growth and plants response to abiotic stress. As shown previously, MYB gene family members in maize and cotton enhanced the tolerance to drought and salt stress by regulating the ABA signaling pathway (Wu et al., 2019; Zhao et al., 2019). R2R3-type MYB transcription factors, *OsMYB91* and *TaSIM*, in rice and wheat had positive effects on salt stress tolerance (Zhu et al., 2015; Yu et al., 2017). The tae-miR319 and tae-miR9666b-3p miRNAs identified in this study were down-regulated under salt stress conditions, whereas, their target genes, traesCS3A02G108000 and traesCS1B02G449200 (*MYB-like* gene) were up-regulated, indicating that tae-miR319 and tae-miR9666b-3p influence the salt tolerance of wheat by regulating the MYB gene.

### tae-miR1131 Enhances Antioxidant Stress Capacity of Wheat by Regulating CYP450

Under normal environmental conditions, reactive oxygen species (ROS) in plants are in a dynamic equilibrium state of generation and elimination. However, under salt stress, plants produce a high concentration of ROS, which accumulates in cells, damages the membrane lipid structure, and affects normal physiological metabolism. CYP450, a multifunctional oxidase, plays a crucial role in plant biosynthesis, metabolism, detoxification, and stress resistance (Li and Wei, 2020). *TaCYP78A3* influences integumentary cell proliferation and thus regulates wheat seed size (Ma et al., 2015). Downregulated *OsCYP707A7* induced a higher ABA content and antioxidant enzyme activity in rice (Cai et al., 2015). *CYP85A1* up-regulation in spinach improved the drought resistance of transgenic tobacco (Duan et al., 2017). We speculated that tae-miR1131 identified in this study enhances the antioxidant stress capacity of wheat by negatively regulating the target genes traesCS7D02G136400 (*CYP450*) and improves the salt tolerance of wheat.

### tae-miR1131 and Novel_72 Co-regulate *POT* Gene to Promote Nutrient Uptake of Wheat

Previous studies demonstrated that wheat tae-miR1131 targets N and P nutrition-related genes that have important roles in wheat N and P nutrient absorption (Kumar et al., 2018). POT is a proton-coupled oligopeptide transporter and is an important nutrient absorption transporter (Newstead, 2017). In the current study, nutrient uptake-related gene TraesCS7B02G312500 (*POT*) was found to be the target gene of tae-miR1131 and predicted Novel_72 miRNAs. It suggests that tae-miR1131 and Novel_72 under salt stress enhance wheat nutrient absorption and salt tolerance by co-regulating *POT* expression.

### tae-miR9774 and tae-miR9668-5p Target Stress Resistance Gene *NB-ARC* for Wheat Salt Tolerance Enhancement

It is known that miR9774 in barley is involved in drought stress (Bakhshi et al., 2017). Besides, tae-miR9774 increases the soil cadmium stress tolerance in wheat plants (Zhou et al., 2019). tae-miR9668-5p improves wheat’s resistance to powdery mildew, leaf rust, and other fungal diseases (Nair et al., 2019). However, only a few studies have explored the salt tolerance of these two miRNAs. NB-ARC family, an important class of stress resistance proteins in plants, plays a vital role in wheat disease resistance (Chandra et al., 2017). Nevertheless, the role of these genes in the salt tolerance aspect of wheat remains unknown. In this study, tae-miR9774 and tae-miR9668-5p were found to be down-regulated under salt stress condition, while their target genes TraesCS1A02G025500, TraesCS7A02G038900, Triticum_aestivum_newGene_26283, and Triticum_aestivum_newGene_26286 (NB-ARC through function prediction) were up-regulated. These results suggest that tae-miR9774 and tae-miR9668-5p regulate the adaptability of wheat to salt stress by negatively regulating the expression of NB-ARC family genes.

### tae-miR1122a Regulates GDSL-Like Lipase to Maintain Lipid Balance in Wheat

Previous studies showed that tae-miR1122a plays a crucial role in the growth and development as well as stress response of wheat. Besides, tae-miR1122a was found to be involved in the anther development of wheat male sterility (Sun et al., 2018). In alkali-tolerant SR4 wheat, tae-miR1122a was found to inhibit ROS accumulation and enhance alkaline stress tolerance by regulating its target gene *Rboh* expression (Han et al., 2018). GDSL esterase also plays a vital role in abiotic stress, pathogen defense, seed development, and lipid metabolism (Tan et al., 2014; Su et al., 2020). However, there is no report about the effect of GDSL esterase on wheat salt tolerance. tae-miR1122a, which was found to be significantly down-regulated in this study under salt stress, could target and negatively regulate the TraesCS7B02G312500 (GDSL-like lipase). Thus, we speculate that tae-miR1122a regulates the lipid balance in wheat in response to the harmful effect of salt stress by regulating GDSL-like lipase.

## Conclusion

In our study, salt-tolerant wheat variety QM6 miRNAs and their target genes in response to salt stress were identified using miRNA and mRNA sequencing. The sequencing data were validated using qRT-PCR. The bioinformatics analysis led to the identification of eight down-regulated miRNAs (tae-miR319, tae-miR9666b-3p, tae-miR1131, tae-miR1131, Novel_72, tae-miR9774, tae-miR9668-5p, and tae-miR1122a) and 11 up-regulated target mRNAs. These mRNAs were found to be involved in transcriptional regulation (MYB), antioxidant stress (CYP450), nutrient uptake (POT), stress tolerance (NB-ARC), lipid balance (GDSL-like lipase), and other biological processes under salt stress. These results indicate that these miRNAs form a complex regulatory network by negatively regulating the expression of their target genes, which play an important role in conferring salt tolerance of the QM6 wheat cultivar. The study enhanced our understanding towards the salt tolerance related miRNAs and their target genes, which can potentially help breeding salt tolerant wheat varieties.

## Data Availability Statement

The datasets presented in this study can be found in online repositories. The names of the repository/repositories and accession number(s) can be found in the article/Supplementary Material.

## Author Contributions

XH and PM: experimental design. ZH, FC, YD, LZ, and XL: experimental performance. XH, ZH, FC, and YD: data analysis. XH and ZH: manuscript writing. HY and JZ: manuscript revision. WM and PM: English language improvement. All authors contributed to the article and approved the submitted version.

## Conflict of Interest

The authors declare that the research was conducted in the absence of any commercial or financial relationships that could be construed as a potential conflict of interest.

## Publisher’s Note

All claims expressed in this article are solely those of the authors and do not necessarily represent those of their affiliated organizations, or those of the publisher, the editors and the reviewers. Any product that may be evaluated in this article, or claim that may be made by its manufacturer, is not guaranteed or endorsed by the publisher.

## Footnotes

^1^ http://www.fao.org/home/en/

^2^ https://cutadapt.readthedocs.io/en/stable/

^3^ https://www.ncbi.nlm.nih.gov/

^4^ https://rfam.org/

^5^ http://www.mirbase.org/

^6^ https://github.com/rajewsky-lab/mirdeep2/releases

^7^ http://www.bioconductor.org/packages/devel/bioc/html/DESeq2.html

^8^ http://www.ccb.jhu.edu/software/hisat/

^9^ http://ccb.jhu.edu/software/stringtie/

^10^ http://pfam.xfam.org/

^11^ http://www.hmmer.org/download.html

^12^ http://omicslab.genetics.ac.cn/psRobot/
