# Supplementary material for: High-Throughput Sequencing-Based Identification of miRNAs and Their Target mRNAs in Wheat Variety Qing Mai 6 Under Salt Stress Condition
Source: Front Genet. 2021 Aug 11;12:724527. doi: 10.3389/fgene.2021.724527 (PMC8385717; doi:10.3389/fgene.2021.724527)
Supplement: Supplementary file 1 [file Table_1.DOCX]

**Supplemental Figure 1.** The volcano plot of known miRNAs. The x-axis represents the expression fold change of miRNA in salt-treated/control samples, the y-axis represents the statistical significance of the miRNA expression change, each scatter represents a miRNA.

**Supplemental Figure 2.** The volcano plot of novel miRNAs. The x-axis represents the expression fold change of miRNA in salt-treated/control samples, the y-axis represents the statistical significance of the miRNA expression change, each scatter represents a miRNA.

**Supplemental Figure 3.** Principal Component analysis (PCA) of gene expression under control or salt treatment. PC1, the first principal component; PC2, the second principal component, n=3. T1: QM6 CK1, T2: QM6 CK2, T3: QM6 CK3, T4: QM6 T1, T5: QM6 T2, T6: QM6 T3.
